# Germline *POT1* Variants: A Critical Perspective on *POT1* Tumor Predisposition Syndrome

**DOI:** 10.3390/genes15010104

**Published:** 2024-01-16

**Authors:** Virginia Andreotti, Irene Vanni, Lorenza Pastorino, Paola Ghiorzo, William Bruno

**Affiliations:** 1 Genetics of Rare Cancers, IRCCS Ospedale Policlinico San Martino, Largo Rosanna Benzi 10, 16132 Genoa, Italy; virginia.andreotti@hsanmartino.it (V.A.); irene.vanni@hsanmartino.it (I.V.); lorenza.pastorino@unige.it (L.P.); paola.ghiorzo@unige.it (P.G.); 2 Department of Internal Medicine and Medical Specialties (DiMI), University of Genoa, V.le Benedetto XV 6, 16132 Genoa, Italy

**Keywords:** POT1, pathogenic variants, germline, melanoma, cancer predisposition, telomeres

## Abstract

The Protection of Telomere 1 (*POT1*) gene was identified as a melanoma predisposition candidate nearly 10 years ago. Thereafter, various cancers have been proposed as associated with germline *POT1* variants in the context of the so-called *POT1* Predisposition Tumor Syndrome (POT1–TPD). While the key role, and related risks, of the alterations in *POT1* in melanoma are established, the correlation between germline *POT1* variants and the susceptibility to other cancers partially lacks evidence, due also to the rarity of POT1–TPD. Issues range from the absence of functional or segregation studies to biased datasets or the need for a revised classification of variants. Furthermore, a proposal of a surveillance protocol related to the cancers associated with *POT1* pathogenic variants requires reliable data to avoid an excessive, possibly unjustified, burden for *POT1* variant carriers. We propose a critical perspective regarding data published over the last 10 years that correlate *POT1* variants to various types of cancer, other than cutaneous melanoma, to offer food for thought for the specialists who manage cancer predisposition syndromes and to stimulate a debate on the grey areas that have been exposed.

## 1. Introduction

**Figure 1:**
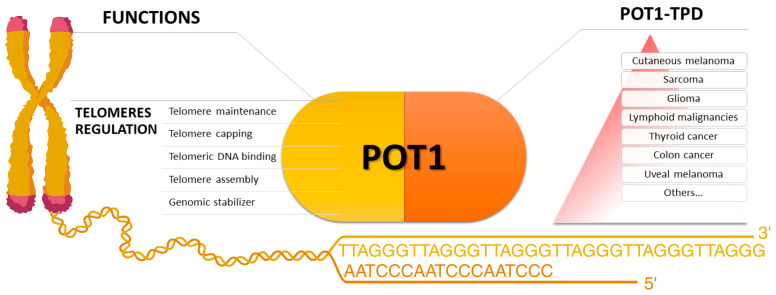
On the left, the main functions in which the protein Protection of Telomere 1 is involved. On the right, a summary of the main cancers proposed as associated with POT1 Tumor Predisposition Syndrome. Cutaneous melanoma is at the top of the pyramid for the consistency of the data supporting its association with the syndrome (created with BioRender.com).

Protection of Telomere 1 (POT1), encoded by a gene on chromosome 7q31.33, is a key subunit of the shelterin telomere binding complex, which protects cells from inappropriate DNA damage response and plays a critical role in telomere regulation. In particular, OB1 and OB2 domains at the N terminus of this essential subunit bind the terminal portions of single-stranded telomeres (ssDNA, TTAGGG repeats) through the interaction with TPP1/ACD, another component of the shelterin complex. The shortening of telomeres occurs at each cell cycle, and POT1 and the shelterin complex protect the ends of chromosomes from instability and regulate their length [1,2]. As an integral part of a complex capable of controlling cellular senescence mechanisms, POT1 plays a key role in telomere homeostasis, while it also acts as a genomic stabilizer [3]. Studies showed that pathogenic variants in the *POT1* gene are associated with the alteration of the telomere length and a subsequent increased risk for cancer. Therefore, it is a gene of interest in studying cancer progression and predisposition syndromes [4,5,6] (see Figure 1).

In 2014, *POT1* was deemed as a novel high-penetrance susceptibility gene to cutaneous melanoma by two independent studies [5,7]. Functional and animal model studies thoroughly investigated the susceptibility variants found in cutaneous melanoma families, but after that, the number of cancers proposed as associated with the clinical spectrum of the *POT1* predisposition tumor syndrome (POT1–TPD) increased [8].

Since more than 50 variants in *POT1* were identified either as germline predisposition alleles, or somatic mutations in different cancers, evidence was collected to consider *POT1* as a relevant gene in the tumorigenesis process [9]. *POT1* has a positive Z-score for both missense and loss-of-function variants, indicating an intolerance to variation [10], hence the low frequency of *POT1* variants. Furthermore, their apparently incomplete penetrance, or evidence from mouse models that they may contribute to tumorigenesis, but that other mechanisms, e.g., p53 deficiency, may be required [11], need to be considered when evaluating the strength of proposed clinical associations.

As aforementioned, germline *POT1* variants were reported as associated with cancers other than melanoma, such as glioma, lymphoid malignancies, colon and thyroid cancer, uveal melanoma, and sarcomas [9]. Hence, a Li–Fraumeni-like surveillance protocol for *POT1* germline variant carriers was also proposed [12], although a standardization on this issue is missing.

Moreover, some doubts related to the nomenclature, pathogenicity classification, or the validity of the clinical association of some variants may be raised, especially after the 2015 American College of Medical Genetics and Genomics (ACMG) classification that, although not flawless [13,14], was implemented in the routine variant classification as the practice of a periodic reassessment.

Our aim here is to discuss the proposed cancer associations, other than cutaneous melanoma, related to the germline variants of *POT1,* reported mostly after 2014, in order to look at strengths, or weaknesses, of the data with a critical perspective.

## 2. Materials and Methods

Through PUBMED, we selected 27 out of 355 papers published from the start of 2014 until the end of July 2023 using as keywords (POT1) AND ((mutation) OR (variant) OR (predisposition) OR (gene) OR (germline) OR (tumor) OR (cancer risk)), excluding reviews and studies not proposing cancer risk correlation with germline variants of *POT1*. This approach has potential limitations derived from unintentional biases, e.g., the chosen keywords, in the selection of the papers. Nevertheless, the relatively small number of actually cited papers may reflect the accuracy in the selection process, focused on the cancer spectrum of POT1–TPD other than cutaneous melanoma.

The studies were grouped according to the proposed clinical association and critically reviewed in the article, possibly including additional articles, or evidence from international databases, regarding the described variants.

All variants are listed in Table 1, along with the updated Human Genome Variation Society (HGVS) nomenclature (in the manuscript’s text, the variants are reported as in the original papers), a revised pathogenicity classification, the proposed cancer association and other details, e.g., the relative position in the folded domain, the allele frequency, and the reference Single Nucleotide Polymorphisms (SNP) ID. Variant data were taken from gnomAD and Franklin (https://franklin.genoox.com/clinical-db/home, accessed on 15 December 2023).

## 3. Results

We identified 49 *POT1* variants reported as associated with cancers other than melanoma. The variants are listed in Table 1 as reported in the cited articles and, when available, with an updated nomenclature and a proposed pathogenicity reclassification compared with the original one. A pathogenicity classification was not reported for 13 variants, of which only one could be classified as a pathogenic variant (PV). The pathogenicity classification was confirmed for five Variants of Unknown Significance (VUSs) and 16 Likely Pathogenic (LP) or PVs. Nine variants classified as PVs, likely pathogenic or deleterious, were reclassified as VUSs. One variant classified as LP was reclassified as likely benign. One VUS was reclassified as benign, another one as LP. One loss-of-function variant was reclassified as a PV. Two variants with a conflicting classification were reclassified as LP and a VUS, respectively.

We grouped articles by the initially proposed cancer association in the subsequent paragraphs reviewing, for each, when available, the critical points summarized below.

- Pathogenicity assessment of the POT1 variants:
  (a) incomplete (only in silico)/not updated;
  (b) absence of functional studies other than not uniformed/standardized procedures for telomere length measurements.
- Reproducibility and consistency of the data:
  (a) finding of a variant in single cases/few families and/or reported only once;
  (b) absence of segregation analysis;
  (c) generic next-generation sequencing approaches or Genome Wide Association Studies (GWAS);
  (d) partial demonstration/evidence for the proposed cancer association with POT1–TPD;
  (e) *POT1* variants as possible by-product of extended testing;
  (f) evaluation of the possible contribution of additional risk factors to the observed cancer histories.
- Variant specific genotype-phenotype correlation
- Recommended improvements:
  (a) need for statistical validation of the proposed cancer association with POT1–TPD through wide and systematic studies;
  (b) novel approaches for pathogenicity assessment.

### 3.1. Glioma

In 2014, a study reported the finding of three variants in three out of 301 glioma families: p.G95C, p.E450X, and p.D617Efs*9 (reported as p.D617Efs*8), the first two segregating in two affected relatives of these small families and with a likely incomplete penetrance [29].

The binding between POT1 and telomeres or TPP1/ACD was predicted to be disrupted. As a functional study, Telomere Length (TL) analysis was performed. As noted in a relative commentary, and its reply, it is not excluded that the finding of the variants could be by chance, and the clinical association lacked further functional validation and reproducibility of the data [40,41].

Interestingly, a study on a native mouse glioma model demonstrated that only p.G95C, a missense variant in the *POT1* OB1 DNA-binding domain, can play a proliferative role in glioma initiation. The authors also suggest that, due to the complexity of gliomagenesis and peculiar effects potentially dependent on *POT1* variants, e.g., sexual dimorphism, further mechanisms should be investigated [37].

On the sidelines, another study suggested a correlation between glioma risk and *POT1*, albeit as a risk factor locus, along with others involved in telomere regulation [15].

### 3.2. Colon Cancer

One study reported that whole-exome (WES) and whole-genome sequencing (WGS) performed on a total amount of 6558 cases of ColoRectal Cancer (CRC) allowed to find in three different cases three *POT1* variants: the aforementioned p.D617Efs*9, p.Arg363Ter, and p.Asn75LysfsTer16 [26].

A subset-based meta-analyses of 204,993 SNPs, among 61,851 cancer cases, reported 13 SNPs as associated with cancer risk. Only the rs116895242 near *POT1* was evaluated as inversely associated with colorectal, ovarian, and lung cancers [42].

An association among seven SNPs in *POT1* and susceptibility to CRC was reported. Still, only one SNP (rs7794637) was previously associated with a cancer predisposition, i.e., to breast cancer, and no further evaluations were performed [16].

### 3.3. Thyroid Cancer

In 2017, the p.K90E variant was identified via WES in a family of eight members affected by cutaneous melanoma. Seven of the eight patients with melanoma carried the variant [27].

The variant segregates with individuals who developed cutaneous melanoma, with a positive family history of endocrine diseases, not only thyroid-related, and is currently classified as likely pathogenic. Two of the relatives were diagnosed with thyroid cancer, one along with cutaneous melanoma, and one along with renal cell cancer, but not cutaneous melanoma, yet their carrier status was not assessed.

In 2020, a WGS study on five families with a history of Non-Medullary Thyroid Cancer (NMTC), multinodular goiter, and thyroid benign nodules found the variant p.V29L in one family [19].

Four family members, who presented papillary thyroid carcinoma, carried the variant, while one out of three family members, with only benign nodules, carried it.

In silico studies predicted a weak binding of the OB1 domain to telomeric ssDNA, also demonstrated by a Chip assay. The binding to TPP1/ACD was not tested.

No significant differences were observed regarding protein expression in HEK293T transfected cells or TL between V29L carriers and not carriers (only in transfected cells).

To date this is the only study reporting the p.V29L variant.

Targeted testing of seven NMTC families found no variant nor in *POT1* or other genes allegedly associated with NMTC susceptibility [43].

In the same year, a study proposed an association between the SNP rs58722976, corresponding to a *POT1* intronic variant, and found in three out of 110 childhood cancer patients, and the risk for subsequent thyroid malignant neoplasm. The SNP was also found in 14 out of 4596 patients who did not develop thyroid cancer. An increased TL was also associated with this variant. However, its low frequency did not allow further studies consolidating the clinical association, e.g., focused on possible ancestry differences and biases regarding the predisposition to thyroid diseases [38].

The variant p.I49Mfs*7 was found in a 65-year-old patient with papillary thyroid carcinoma, cutaneous melanoma, and splenic marginal zone lymphoma. The variant was assessed as germline by next-generation sequencing on skin fibroblasts and once reported in a patient with glioma as a putative predisposition variant [24]. Further personal and family history information was unavailable, and no functional studies were performed [25].

### 3.4. Uveal Melanoma

A few genes, e.g., *BAP1* and *MDB4*, are the only established genes correlated to hereditary and syndromic forms of Uveal Melanoma (UM) [44,45].

A study reported two deleterious variants in two out of 20 UM patients [28]. Both patients also developed cutaneous melanoma. However, the patients were from Australia, the country with the highest melanoma incidence, and melanoma was diagnosed after 65 years of age. Segregation and TL measurement, as functional analysis, were evaluated for both cases. While the first variant, p.Q94Rfs*13 (reported as p.Q94Rfs*12), is predicted as likely pathogenic, the second one, written as c.1851_1852del, is the same discussed above in association with glioma risk, or reported in a CRC cohort (p.D617Efs*9).

### 3.5. Sarcoma

A peculiar scenario of variant-specific phenotype is the one described for the risk of sarcomas, e.g., cardiac angiosarcoma, in correlation with the founder variant p.R117C, identified in four TP53-negative Li–Fraumeni-Like (LFL) Spanish families [31].

In a subsequent study, the authors identified four additional variants predicted to be damaging in two out of 10 LFL families probands with angiosarcoma (p.Gln301*, p.T497K, being reported as T497L) and in two out of 30 sporadic cardiac angiosarcoma cases (p.R116C, c.547-1G>A). In contrast, one sporadic case carried the p.R117C variant. Of note, no *POT1* variant was found in 24 LFL families without angiosarcoma cases [30].

Above all, data from in vitro studies confirmed in silico prediction about p.R117C, in terms of reduced telomeres, TPP1/ACD binding of POT1, and abnormal TL.

In 2022, a further convincing study observed the same effect on TL for this variant and the development of angiosarcomas in the knock-in mice model [32].

A European WES and targeted sequencing study on 1244 patients with osteosarcoma led to the identification of likely pathogenic (c.124G>T, p.Gln376Arg, c.205C>T, D224N) and pathogenic variants (c.1851_1852delTA) in *POT1*, among a few unexpected genes (*CDKN2A*, *MEN1*, *VHL*, *APC*, *MSH2*, *ATRX*), other than in *TP53*. However, functional analysis for the specific variants was not performed, and the authors also declared some limitations, such as the lack of family history assessment and methodological biases [23].

On a side note, a WES study identified the p.P35L variant in one out of 13 probands of LFL families. The variant is not reported in any database, and its pathogenicity is predicted by in silico structural analysis [20].

### 3.6. Lymphoid Malignancies

In 2013, a GWAS, integrated with metanalyses, mapped new susceptibility loci for Chronic Lymphocytic Leukemia (CLL) to 7q31.33 and proposed an association between the *POT1* 3′UTR SNP rs17246404 and a small CLL increased risk [39]. In 2016, a WES study from the same authors identified four deleterious *POT1* variants, each segregating in four out of 66 CLL families [22]. p.Tyr36Cys, previously reported at somatic level in CLL cases [46,47], was predicted by a structural prediction tool to impact POT1 and telomeric DNA affinity, whereas c.1164-1G>A, p.Gln358SerFTer13, and p.Gln376Arg were predicted to impair POT1-TPP1/ACD interaction. For the latter variant, an analysis of 1083 cases vs. 5854 controls showed a higher risk of CLL (3.61-fold). Nonetheless, while being in an evolutionarily conserved residue, it was predicted to not affect the protein stability and was not in linkage disequilibrium with SNP rs17246404. No significant difference in TL was observed for all the variants.

In 2018, a WES study identified two *POT1* variants in two out of 41 Hodgkin lymphoma families, D224N and Y36H, the second one seemingly with incomplete penetrance (one carrier was unaffected). For both variants, an increased TL was observed in human cells [21]. A variant similar to Y36H, Y36N, was previously studied, and the binding to the telomeric overhang was not compromised [4], in contrast to another study that identified the same variant as somatic in CLL cases, and demonstrated an impact on telomeric binding [46]. Of note, the susceptibility hypothetically underlying a familial clustering of Hodgkin lymphoma is still far to be uncovered, and other rare variants segregated in the two families. Hence, while a contribution of deleterious *POT1* variants may not be ruled out, other genes may be equally worth investigating.

The D224N was aforementioned among the variants found through a WES performed on osteosarcoma cases and reported as segregating in a family with a variety of lymphoid malignancies (Hodgkin lymphoma, Myeloid Chronic Leukemia, Follicular lymphoma) and with a proband who also developed cutaneous melanoma, renal cell carcinoma, colon cancer, and prostate cancer. The father developed a similar cluster of cutaneous melanoma and lymphoid malignancies, although from 62 to 76 years of age. Neoplastic tissues for further studies were not available, and other possible risk factors, e.g., lifestyle, sun exposure, environment, and the median age of diagnosis in the general population, were not reported or highlighted [35].

In 2021, the p.Q199* germline variant was identified in only one child with Acute Myeloid Leukemia for which evidence of pathogenicity was found at cell, protein expression, and mRNA levels [34].

An exome analysis found three *POT1* variants in as many multiple myeloma patients out of 128 patients: c.458T>A, c.1594G>C, and c.547-1G>A [17].

The first one was found in a patient also diagnosed with thyroid cancer at 36 years and whose father, affected by multiple myeloma, was not a carrier of the variant. The second one was found in a proband just diagnosed with osteosarcoma at 20 years, multiple myeloma at 66, and acute myeloid leukemia at 70 and previously identified in a cutaneous melanoma family [5]. The third variant, also found in the proband’s mother with a diagnosis of multiple myeloma, is the same described in a family with angiosarcoma [30]. Multiple myeloma was not reported in both of the cutaneous melanoma or sarcoma families. The same study reported the variant c.1A>G (p.Met1Val) in a patient diagnosed with essential thrombocythemia and Monoclonal Gammopathy of Undetermined Significance at 77 and papillary thyroid carcinoma at 78 years of age.

A recent study on a case series of 3323 patients with different diagnoses of myeloproliferative neoplasm identified several somatic, germline, and putative germline *POT1* variants. Thirteen variants were established as germline, and for four patients, a history of cancers, allegedly associated with *POT1* variants, is reported (cutaneous melanoma, glioma, thyroid cancer). Eight of these variants are actually deemed as a VUS or as benign [18].

## 4. Discussion

Since the identification of *POT1* as a novel gene implied in rare hereditary forms of cutaneous melanoma, many studies proposing to add other cancers to the clinical spectrum of the so-called POT1–TPD have been published.

Although the association with a higher risk of cutaneous melanoma is established, and it seems reasonable to hypothesize that *POT1* variants may confer a wider cancer risk, given the biological processes in which it is involved, a convincing demonstration of the clinical association between the variants and the observed cancers to be possibly included in the spectrum of POT1–TPD is often missing. First, assessment of the pathogenicity of the candidate variants is often incomplete, and many of them need to be revised according to ACMG recommendations.

The main issue can be traced back to the discovery of a variant in a single case [33], where there was no co-segregation analysis; i.e., only the proband was tested, in a few families or from extended but generic GWAS [36] supporting the association.

Nevertheless, other sticking points merit a collective discussion.

Among these, the finding of a variant, actually classified as a VUS or as benign (see Section 3 and Table 1), which was reported only once, has a further impact on the reproducibility and consistency of the data.

Moreover, functional studies, in vitro and in vivo, other than telomere length assessment, are scarce.

While the impact of *POT1* variants on telomere length is the subject of interest in many studies, some authors have shown it as the sole evidence of a variant pathogenicity, due to its correlation with cancer susceptibility [1,48]. This limitation is also emphasized by the lack of uniform or standardized procedures in the different studies. Furthermore, as for some reported clinical associations, e.g., glioma, variants in other telomere length-associated loci represented a possible risk factor (also in haplotype blocks) [15], or the telomere length may not have been the only parameter to be evaluated to establish the biological impact of a *POT1* variant, e.g., effect on specific gene pathways [49].

A study proposing a clinical association should consider the hypothesis that the observed *POT1* variant was found by chance, as a by-product from extended genetic testing, e.g., WES or WGS. Other confounding or additional risk factors that might contribute to the observed cancer histories, especially when the observed age of diagnosis of a neoplasia is in the range of the general population, should be considered.

The scenario of a variant-specific genotype–phenotype correlation concerning the c.948C>T variant, for which many functional evidences are available, leaving aside the uncertainties in translating the results from animal models to humans, is of interest.

The sparseness of data regarding *POT1* variants, and the spectrum of POT1–TPD, also points out the need for wide and systematic studies, including statistical validations, one of the strongest methods of confirming a “cancer risk”, as recently performed in a retrospectively analysis of the results derived from multigene panel testing applied on a large cohort of patients (13.315) evaluated for various cancer predisposition syndromes. While the study consolidated the association with cutaneous melanoma risk, a statistically significant increased risk for sarcoma was also observed [8].

It may be untimely to suggest a “Li–Fraumeni-Like” surveillance protocol for carriers of any pathogenic variant in *POT1*, except for some specifically sarcoma-associated variants, in order to avoid the burden of oversurveillance. Still, the inclusion of sarcomas in POT1–TPD could be a critical topic of discussion in the future on the basis of the accrual of further evidence.

In conclusion, a systematic approach to assess the pathogenicity of the variants, and their clinical significance, is necessary to accurately define the clinical spectrum of POT1-PTD and to discuss, with solid data, the protocol of surveillance for *POT1* variant carriers. Novel methodological approaches such as saturation genome editing [50,51], as well as the appropriate collection of statistically significant data from larger cohorts, instead of few families or single patients who may carry private variants, will definitively be of help.

## Figures and Tables

**Table 1:** The table includes the *POT1* variants described in the selected literature with the reported cancer association other than melanoma, updated HVGS nomenclature (*POT1* reference NM_015450), a proposed pathogenicity classification according to ACMG guidelines, and, if different, the reported classification when available. Where applicable, the relative position in the folded domain, allele frequency, and reference SNP ID are reported. Variant data were taken from gnomAD and Franklin (https://franklin.genoox.com/clinical-db/home, accessed on 15 December 2023).

			*POT1* Variants Revised						
Reported Cancer Associated with POT1–TPD	chr7 Position	*POT1* Variants as Reported	cDNA	Protein	Proposed vs. Original Classification	Dom	A.F.	Reference SNP ID	Ref.
glioma	124,917,495	rs56356267	c.-226-1849T>C		B/-		0.2186000	rs56356267	[15]
glioma	124,914,213	rs59294613	c.-154+1361G>T		B/-		0.2744000	rs59294613	[15]
colon	124,900,818	rs7782354	c.-153-2444G>A		B/-		0.5653000	rs7782354	[16]
colon	124,900,787	rs4383910	c.-153-2413T>G		B/-		0.5464000	rs4383910	[16]
multiple myeloma	124,897,173	c.1A>G; p.Met1Val	c.1A>G	p.(Met1Val)	LP		0.0000016	rs1584510207	[17]
myeloproliferative disorders	124,892,382	c.10-2A>C	c.10-2A>C		P/LP		na	rs1554434788	[18]
thyroid	124,892,305	p.V29L	c.85G>C	p.(Val29Leu)	VUS/P	OB1	na	na	[19]
sarcoma	124,892,286	p.P35L	c.104C>T	p.(Pro35Leu)	VUS/P	OB1	na	na	[20]
lymphoma	124,892,284	Y36H	c.106T>C	p.(Tyr36His)	VUS /deleterious	OB1	0.0000007	na	[21]
leukemia	124,892,283	p.Tyr36Cys	c.107A>T	p.(Tyr36Cys)	VUS /deleterious	OB1	0.0000007	na	[22]
osteosarcoma	124,892,266	c.124G>T	c.124G>T	p.(Asp42Tyr)	VUS/LP	OB1	0.0000054	rs1358511734	[23]
cutaneous melanoma, thyroid, lymphoma	124,871,018	p.I49Mfs*7; c.147delT	c.147del	p.(Ile49Metfs*7)	P	OB1	0.0000031	rs1064794328	[8,24,25]
osteosarcoma	124,870,961	c.205C>T	c.205C>T	p.(Leu69Phe)	VUS/LP	OB1	0.0000016	rs905571705	[23]
colon	124,870,941	p.Asn75LysfsTer16	c.224dup	p.(Asn75Lysfs*16)	P	OB1	na	na	[26]
myeloproliferative disorders	124,870,933	c.T233C; p.I78T	c.233T>C	p.(Ile78Thr)	LP/VUS	OB1	0.0000087	rs947005337	[8,18]
cutaneous melanoma, thyroid	124,863,628	c.A268G; p.K90E	c.268A>G	p.(Lys90Glu)	LP	OB1	0.0000131	rs1554427012	[27]
uveal melanoma	124,863,613	p.Q94Rfs*12	c.281_282del	p.(Gln94Argfs*13)	P/LP	OB1	0.0000014	rs1397398300	[28]
glioma	124,863,613	p.G95C	c.283G>T	p.(Gly95Cys)	VUS/-	OB1	0.0000032	rs797045168	[29]
angiosarcoma	124,863,549	c.946C>T; p.P116L	c.347C>T	p.(Pro116Leu)	LP	OB1	0.0000032	rs1554426966	[30]
angiosarcoma	124,863,547	p.R117C	c.349C>T	p.(Arg117Cys)	LP	OB1	0.0000014	rs780936436	[30,31,32]
myeloproliferative disorders	124,863,447	c.T449C; p.L150S	c.449T>C	p.(Leu150Ser)	VUS	OB2	0.0000470	rs918544320	[18]
multiple myeloma	124,863,438	c.458T>A; p.Leu153Ter	c.458T>A	p.(Leu153*)	LP	OB2	na	na	[17]
myeloproliferative disorders, papillary cystadenoma of the epididymis	124,863,370	c.526G>A; (p.Gly176Arg)	c.526G>A	p.(Gly176Arg)	VUS	OB2	0.0000155	rs774576173	[18,33]
angiosarcoma, multiple myeloma	124,859,113	c.547-1G>A	c.547-1G>A		LP		0.0000054	na	[17,30]
leukemia	124,859,064	p.Q199*	c.595C>T	p.(Gln199*)	P/LOF	OB2	na	na	[34]
myeloproliferative disorders, lymphoma, osteosarcoma	124,858,989	D224N	c.670G>A	p.(Asp224Asn)	VUS/LP /deleterious /P/VUS	OB2	0.0001701	rs202187871	[18,21,23,35]
myeloproliferative disorders	124,853,138	c.G703A; p.V235I	c.703G>A	p.(Val235Ile)	VUS	OB2	0.0000103	rs753638532	[18]
myeloproliferative disorders	124,853,088	c.G753A; p.M251I	c.753G>A	p.(Met251Ile)	VUS	OB2	0.0000020	rs1172142052	[18]
colon	124,851,984	rs7784168	c.870-33A>G		B/-		0.3171738	rs7784168	[16]
angiosarcoma	124,851,920	p.Gln301*	c.901C>T	p.(Gln301*)	LP		na	na	[30]
not specified	124,846,992	c.954_955DUPTG;p.G319VFS*9	c.954_955dup	p.(Gly319Valfs*9)	P		0.0000019	rs1436069277	[8]
myeloproliferative disorders, lung	124,846,971	p.V326A	c.977T>C	p.(Val326Ala)	B/VUS/-	TPP1	0.0002260	rs75932146	[18,36]
colon, thyroid	124,846,926	rs7794637	c.1006+16A>T		VUS/-		na	rs7794637	[16]
leukemia	124,842,898	p.Gln358SerfsTer13	c.1071dup	p.(Gln358Serfs*13)	P	TPP1	0.0000503	rs750470470	[8,22]
colon	124,842,883	p.Arg363Ter	c.1087C>T	p.(Arg363*)	P/-	TPP1	0.0000286	rs756198077	[8,26]
myeloproliferative disorders	124,842,862	c.1107delT; p.Y369*	c.1107del	p.(Tyr369*)	P	TPP1	0.0000197	rs1487490244	[8,17]
leukemia, osteosarcoma	124,842,843	p.Gln376Arg	c.1127A>G	p.(Gln376Arg)	VUS/LP /deleterious	TPP1	0.0005205	rs143635917	[22,23]
colon	124,841,191	rs3815221	c.1164-13C>T		B/-		0.3981377	rs3815221	[16]
myeloproliferative disorders, leukemia	124,841,179	c.1164-1G>A	c.1164-1G>A		P/LP/P		0.0000398	rs866612394	[8,18,22]
myeloproliferative disorders	124,841,062	c.A1280C; p.K427T	c.1280A>C	p.(Lys427Thr)	VUS	TPP1	0.0000096	rs1187946222	[18]
glioma	124,840,994	p.E450X	c.1348G>T	p.(Glu450*)	P/LP	TPP1	na	rs797045169	[29,37]
thyroid	124,840,584	rs58722976	c.1369+389T>C		B/-		0.0135800	rs58722976	[38]
angiosarcoma	124,835,294	c.1490C>A; p.T497L	c.1490C>A	p.(Thr497Lys)	LB/LP	TPP1	0.0000186	rs879897044	[30]
multiple myeloma	124,829,254	c.1594G>C; p.Ala532Pro	c.1594G>C	p.(Ala532Pro)	VUS/LP	TPP1	0.0000042	rs537377921	[17]
colon	124,829,213	rs10263573	c.1594+41T>G		VUS/-		0.3981215	rs10263573	[16]
myloproliferative disorders, glioma, colon, uveal melanoma, osteosarcoma	124,824,014	p.D617Efs*8	c.1851_1852del	p.(Asp617Glufs*9)	LP/-/LP/VUS/P	TPP1	0.0000628	rs758673417	[8,18,23,26,28,29,37]
myeloproliferative disorders	124,823,997	c.A1870G; p.I624V	c.1870A>G	p.(Ile624Val)	VUS	TPP1	0.0000118	rs1410012081	[18]
leukemia	124,822,607	rs17246404	c.*1355G>T		LB/-		0.2018000	rs17246404	[39]
colon	124,822,601	rs76436625	c.*1361A>G		B/-		0.1559000	rs76436625	[16]

A.F.: Allele Frequency; ACMG: The American College of Medical Genetics and Genomics; B: Benign; gnomAD: Genome Aggregation Database; Dom: domain; HVGS: Human Genome Variation Society; LB: Likely Benign; LOF: Loss-Of-Function variant; LP: Likely Pathogenic; na: not available; P: Pathogenic; SNP: Single Nucleotide Polymorphism; VUS: Variant of Unknown Significance.

## Data Availability

Data sharing not applicable.
